# Effect of Workplace Noise on Hearing Ability in Tile and Ceramic Industry Workers in Iran: A 2-Year Follow-Up Study

**DOI:** 10.1155/2013/923731

**Published:** 2013-12-22

**Authors:** Mehrdad Mostaghaci, Seyyed Jalil Mirmohammadi, Amir Houshang Mehrparvar, Maryam Bahaloo, Abolfazl Mollasadeghi, Mohammad Hossein Davari

**Affiliations:** ^1^Department of Occupational Medicine, School of Medicine, Shahid Sadoughi University of Medical Sciences, Shahid Rahnemoun Hospital, Farrokhi Avenue, Yazd 89138-14389, Iran; ^2^Industrial Disease Research Center, Shahid Sadoughi University of Medical Sciences, Shahid Rahnemoun Hospital, Farrokhi Avenue, Yazd 89138-14389, Iran

## Abstract

*Introduction*. Noise as a common physical hazard may lead to noise-induced hearing loss, an irreversible but preventable disorder. Annual audiometric evaluations help detect changes in hearing status before clinically significant hearing loss develops. This study was designed to track hearing threshold changes during 2-year follow-up among tile and ceramic workers. *Methods*. This follow-up study was conducted on 555 workers (totally 1110 ears). Subjects were divided into four groups according to the level of noise exposure. Hearing threshold in conventional audiometric frequencies was measured and standard threshold shift was calculated for each ear. *Results*. Hearing threshold was increased during 2 years of follow-up. Increased hearing threshold was most frequently observed at 4000, 6000, and 3000 Hz. Standard threshold shift was observed in 13 (2.34%), 49 (8.83%), 22 (3.96%), and 63 (11.35%) subjects in the first and second years of follow-up in the right and left ears, respectively. *Conclusions*. This study has documented a high incidence of noise-induced hearing loss in tile and ceramic workers that would put stress on the importance of using hearing protection devices.

## 1. Introduction

Noise is the most common physical hazard in the industrial workplaces. A report from the European Union mentions that about 28% of workers are exposed to noise level approximately between 85 and 90 dBA [1]. The most common health problem due to exposure to noise is noise-induced hearing loss (NIHL), an irreversible but preventable disorder [2]. NIHL is the second most common form of acquired hearing loss, after presbycusis [3, 4], and is a major concern for workers' health in different countries [4–6].

In the industrial settings, when noise exceeds permissible levels, hearing conservation program should be started, which contains hearing evaluation and some other activities.

In 1996 National Institute of Occupational Safety and Health (NIOSH) reported that about 30 million workers in USA are exposed to loud noise which can lead to hearing loss [7]. It is estimated that 10 million workers suffer from NIHL in USA [8].

NIHL is typically a bilateral and symmetric hearing loss with a notch of the audiogram at 3, 4, or 6 kHz and a recovery at 8 kHz as the first sign [4]. This hearing impairment may be aggravated if exposure to noise is continued [9, 10].

Prevention of deafness and hearing impairment (PDH), a WHO program, is especially planned for developing countries due to lack of accurate population-based studies about the prevalence and causes of deafness and hearing loss [11–13]. Occupational Safety and Health Administration (OSHA) requires that all workers exposed to noise more than 85 dBA be screened for NIHL annually [14].

Standard threshold shift (STS) is defined as a 10 dB or more change in average hearing threshold at 2000, 3000, and 4000 Hz. So, even if the audiogram is not abnormal, positive STS is important to find those workers susceptible to hazardous effects of noise on hearing [2, 3, 15].

Annual audiometric evaluations help detect changes in hearing status before clinically significant hearing loss develops [16]. Recently, other methods such as extended high-frequency audiometry and otoacoustic emissions are introduced for early diagnosis of NIHL [17, 18].

Hong found a prevalence of 60% for hearing loss among construction workers which was directly related to work experience. They found left ear to be more sensitive to noise. Workers who used hearing protection devices (HPDs) showed lower frequency of hearing loss than others. They did not find a typical notch at 4 or 6 kHz [4].

In a large study in The Netherlands they assessed the effect of duration of noise and noise level on the frequency of NIHL and found that duration of exposure is more important in NIHL causation than noise level [10]. In another study in a steel rolling mill they found that 56.8% of workers in their worse ear and 28.2% in their better ear suffered from hearing loss and noise level was between 49 and 93 dBA [19].

Other studies in different parts of the world have assessed occupational hearing loss in different industries (Morata et al. 1997 in printing industry [20], Bhattacharya et al. 1990 in a pharmaceutical company [21], and Shaikh 1996 in a polyester fiber plant [22]).

In the tile and ceramic industry, because of some machinery, equipment, and tools, hazardous noise is frequently observed. Tile and ceramic industry is one of the main industries in Iran, and Yazd, a central province, owns the largest numbers of tile and ceramic producing factories in which workers are subject to NIHL. We could not find a study on NIHL in tile and ceramic industry. So this study was designed to track hearing threshold changes during 2 years of follow-up among tile and ceramic workers.

## 2. Materials and Methods

This was a follow-up study conducted on 594 workers from 5 tile and ceramic factories in Yazd, a central province of Iran. Factories were selected by simple random sampling from all tile and ceramic factories in Yazd (*n* = 29). In each factory subjects were selected by simple random sampling from different jobs. Each factory had 14 different job titles including glazing, glaze-making, forming, ball mill, spray drying, mixing and grinding, packing and loading, mechanic, forklift driving, warehouse, firing, printing, service, and office. These 14 subgroups were merged to produce 4 major groups according to noise level. Group 1 (noise level = 75–92 dBA, 8 h time-weighted average (TWA) = 86.4 dBA) includes glazing, glaze-making, forming, packing and loading, forklift driving, firing, printing, and service; group 2 (noise level = 85–101 dBA, 8 h-TWA= 92.6 dBA) includes mixing and grinding, ball mill, and spray drying; group 3 (noise level: 65–101 dBA, 8 h-TWA = 82.3 dBA) includes mechanics; and control group (noise level: lower than 75 dBA) includes warehouse and office workers.

Those with previous history of acoustic trauma, congenital hearing loss, and ototoxic drug consumption and age more than 50 years were excluded from the study. The workers irregularly used hearing conservation devices (ear plugs).

Noise level was extracted from the result of measurements routinely performed in the factories by industrial hygiene incorporations and was presented as time-weighted average (TWA) for an eight-hour shift.

Audiometry was performed for the subjects (using clinical audiometer: AC40, Interacoustic, Denmark, headphone: TDH39) in an acoustic chamber meeting the criteria of ANSI 2004 [23] after at least 16 hours abstinence from noise. The audiologist who performed the tests (baseline and follow-up tests) was the same. Hearing threshold in conventional audiometric frequencies (i.e., 250, 500, 1000, 2000, 3000, 4000, 6000, and 8000 Hz) was measured. Frequencies of 3000, 4000, and 6000 Hz were considered as the frequencies with the highest susceptibility to noise so the mean change (10 dB or more increase in the hearing threshold at these frequencies) was calculated for each job category. STS was calculated for each ear as well. Hearing loss at each frequency was defined as hearing threshold higher than 20 dB. During follow-up, 39 subjects changed their job so were not available for follow-up.

Data was analyzed by SPSS (ver. 18) using Student's *t*-test, chi-square test, and ANOVA. A *P* value of less than 0.05 was taken as the level of significance. An informed consent was obtained from each participant. This study was approved by the ethics committee of Shahid Sadoughi University of Medical Sciences.

## 3. Results

After considering exclusion criteria and subjects who were lost from follow-up, 555 tile and ceramic workers entered the study (totally 1110 ears).  Table 1 shows demographic data of all workers in each job category.

**Figure 1:**
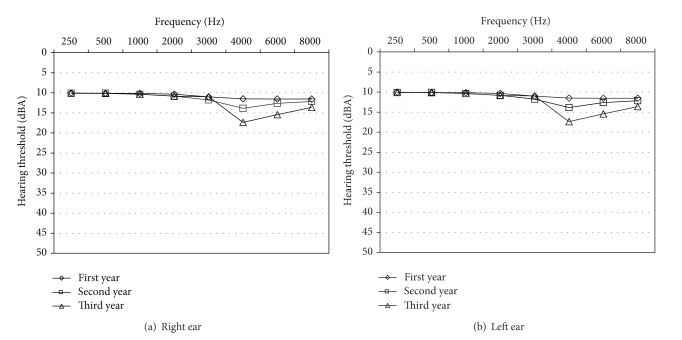
Trend of hearing threshold change in right and left ears in different years of evaluation among all subjects.

Mean hearing threshold was measured at each audiometric frequency.  Figure 1 compares the mean hearing threshold at different frequencies in each ear.  Table 2 shows the prevalence of abnormal threshold (>20 dB) in different frequencies among different job categories. Mean threshold change at 3000, 4000, and 6000 Hz was calculated for each job category which is presented in Table 3.

**Figure 2:**
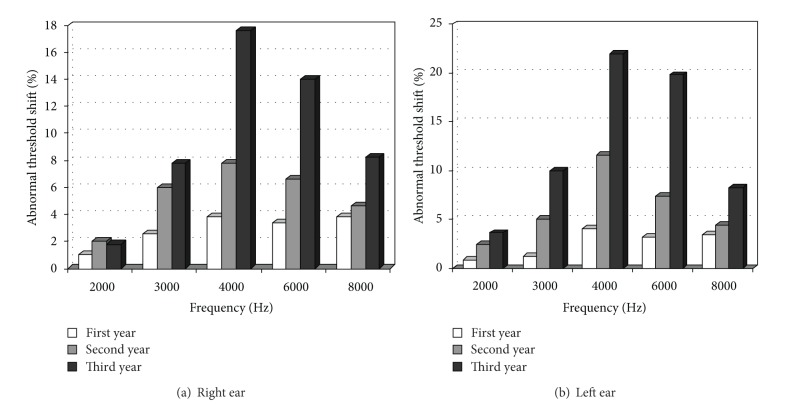
Percentage of abnormal hearing threshold (>20 dB) in different frequencies in right and left ears in different years of follow-up.

Percentage of abnormal threshold in different frequencies in each ear is shown in Figure 2.

A number of subjects showed STS after first and second year of follow-up. STS was observed in 13 (2.34%) and 49 (8.83%) subjects in the first and second years of follow-up in the right ear and in 22 (3.96%) and 63 (11.35%) subjects in the first and second years of follow-up in the left ear.

## 4. Discussion

Noise as a common physical exposure in many industrial workplaces may lead to various health effects, especially NIHL. In this study we evaluated hearing threshold shift in a 2-year follow-up among tile and ceramic workers. The population which we studied was a young population with exposure to continuous noise during their eight hour work shift. Most workers in different parts of tile factories are exposed to noise level higher than ACGIH (American Conference on Governmental Industrial Hygienists) TLV (Threshold Limit Value), that is, 85 dBA. The exposure to noise was not significantly changed during two years of follow-up. To the best of knowledge, this was the first follow-up study for finding hearing loss trends in tile workers in our country. In other countries we could not find similar studies on tile and ceramic workers.

In the factories which were evaluated in this study, Hearing Conservation Program (HCP) according to OSHA is installed but not completely, so annual noise monitoring and annual audiometric tests are mandatory for the workers, but hearing conservation devices are not used regularly and workers are not trained accordingly [24]. The use of HPD was recorded according to the workers' self-report which is not reliable [25–27].

How the workers used HPD is also another important factor that affects the true exposure to noise which could not be evaluated in this study [28, 29].

In this study, the workers were categorized according to the exposure to noise which was evaluated environmentally, so the real exposure of each worker is probably different from that of another worker in the same job [30].

In the current study, although the mean hearing thresholds at all frequencies were in the normal range, a considerable number of workers suffered from NIHL, and its frequency was significantly increased during the follow-up period (after 2 years). We found that hearing loss was significantly higher in the workers exposed to noise than in the control group consistent with the findings of Neitzel et al. [31] and Ologe et al. [19], Leensen et al. [10], Ahmed et al. [32], Osibogun et al. [33], and Shakhatreh et al. [34]. We found the highest frequency of threshold change in the workers with the highest exposure to noise who were working in mixing, grinding, and ball mill.

Hearing loss was most commonly seen at 4000 Hz consistent with many other previous studies [4]. 6000 Hz and 3000 Hz were the second and third frequencies affected which is typical for NIHL to affect frequencies higher than 3000 Hz [4, 10]. So in most cases of NIHL, the affected people are not aware of their impairment, because low audiometric frequencies are much more important for daily conversation [4, 35].

Low audiometric frequencies were affected in a few subjects. It is known that low audiometric frequencies are less susceptible to noise and are affected later than high frequencies, and our study subjects were mostly young persons with work experience less than 15 years. We found that the hearing threshold was clearly increased during follow-up time at these frequencies and lower frequencies were approximately intact even after 2 years of follow-up. We found a higher prevalence of hearing loss in left ear than right ear consistent with Hong [4], Ross et al. [15], Marvel et al. [36], Pirila et al. [37], and Simpson et al. [38], although the exact mechanism of higher involvement of left ear is not understood yet.

We found that about 3% and 10% of subjects suffered from STS after 1 and 2 years follow-up, respectively. The change in mean threshold of 3000, 4000, and 6000 Hz (frequencies most affected by noise) was significantly higher than STS which is measured by the change in mean threshold of 2000, 3000, and 4000 Hz.

One of the typical early signs of NIHL is a V-shaped notch which is mostly seen at the most sensitive frequencies to noise (i.e., 3000, 4000, or 6000 Hz) [39]. We found this typical notch in a considerable number of workers but Hong who evaluated NIHL among construction workers did not find this sign [4].

This study had some limitations: some workers were transformed to another job inside the factory during follow-up period, but we assessed them as the members of the previous job. The number of female workers was low so we could not compare the results between males and females. There were 14 job titles in the factories, but in order to analyze the data we assessed them in four groups, so the workers in each group were exposed to a range of noise level. The workers used HPDs irregularly, so we could not analyze the data regarding HPDs use.

## 5. Conclusion

This study showed a high frequency of hearing loss in tile and ceramic workers in spite of the obligation by health systems to install hearing conservation program.

## Figures and Tables

**Table 1:** Demographic properties of all workers in each job category.

	Group	Mean	SD	*P* value
Age (year)	Control	33.50	7.34	0.719
	1	33.50	6.70	
	2	32.66	7.06	
	3	33.99	6.91	

Work experience (year)	Control	7.94	4.46	0.668
	1	7.91	3.83	
	2	8.33	3.85	
	3	8.45	3.68	

Height (cm)	Control	172.23	8.81	0.110
	1	173.55	6.76	
	2	174.98	5.44	
	3	175.14	5.47	

Weight (Kg)	Control	74.16	11.02	0.194
	1	76.72	14.09	
	2	79.08	12.35	
	3	78.05	12.91	

**Table 2:** Number of subjects with hearing loss in the 1st, 2nd, and 3rd years of evaluation at each frequency considering job category.

		Frequency (Hz)													
	Study group	500		1000		2000		3000		4000		6000		8000	
		Right	Left	Right	Left	Right	Left	Right	Left	Right	Left	Right	Left	Right	Left
		Subjects with hearing loss (*n*, %)													
1st year	Control	0 (0)	0 (0)	0 (0)	0 (0)	0 (0)	0 (0)	0 (0)	0 (0)	0 (0)	0 (0)	0 (0)	0 (0)	0 (0)	0 (0)
	1	0 (0)	0 (0)	0 (0)	0 (0)	4 (2.03)	0 (0)	8 (4.06)	0 (0)	9 (4.57)	9 (4.57)	13 (6.59)	10 (5.07)	12 (6.09)	8 (4.06)
	2	3 (2.67)	3 (2.67)	3 (2.67)	3 (2.67)	0 (0)	0 (0)	4 (3.92)	4 (3.92)	12 (11.76)	12 (11.76)	2 (1.96)	4 (3.92)	0 (0)	6 (5.88)
	3	0 (0)	0 (0)	0 (0)	0 (0)	1 (0.69)	4 (2.78)	3 (2.08)	4 (2.78)	3 (2.08)	4 (2.78)	3 (2.08)	4 (2.78)	7 (4.86)	6 (4.16)

	*P* value	<0.001	<0.001	<0.001	<0.001	0.18	0.009	0.15	0.013	<0.001	<0.001	0.007	0.106	0.005	0.113

2nd year	Control	0 (0)	0 (0)	1 (0.98)	0 (0)	2 (1.78)	2 (1.78)	4 (3.57)	4 (3.57)	5 (4.46)	4 (3.57)	4 (3.57)	5 (4.46)	2 (1.78)	0 (0)
	1	0 (0)	0 (0)	0 (0)	0 (0)	6 (3.04)	2 (1.02)	9 (4.57)	4 (2.03)	15 (7.61)	15 (7.61)	14 (7.11)	17 (8.63)	12 (6.09)	13 (6.59)
	2	3 (2.67)	3 (2.67)	5 (4.46)	6 (5.36)	4 (3.92)	2 (1.96)	26 (25.49)	20 (19.61)	28 (27.45)	43 (42.16)	14 (13.72)	12 (17.64)	4 (3.92)	4 (3.92)
	3	0 (0)	0 (0)	0 (0)	0 (0)	1 (0.69)	7 (4.86)	4 (2.78)	7 (4.86)	5 (3.47)	17 (11.81)	8 (5.56)	7 (4.86)	8 (5.56)	7 (4.86)

	*P* value	<0.001	<0.001	<0.001	<0.001	0.33	0.12	<0.001	<0.001	<0.001	<0.001	0.026	0.11	0.34	0.053

3rd year	Control	0 (0)	0 (0)	1 (0.98)	0 (0)	2 (1.78)	2 (1.78)	4 (3.57)	4 (3.57)	5 (4.46)	4 (3.57)	4 (3.57)	5 (4.46)	2 (1.78)	1 (0.89)
	1	0 (0)	0 (0)	0 (0)	0 (0)	6 (3.04)	4 (2.03)	12 (6.09)	8 (4.06)	17 (8.63)	26 (13.19)	18 (9.14)	21 (10.66)	20 (10.15)	18 (9.14)
	2	4 (3.57)	4 (3.57)	5 (4.64)	7 (6.25)	8 (7.84)	2 (1.96)	30 (29.41)	37 (36.27)	72 (70.59)	85 (83.33)	33 (32.35)	47 (46.08)	12 (17.76)	9 (8.82)
	3	0 (0)	0 (0)	0 (0)	0 (0)	1 (0.69)	12 (8.33)	11 (7.64)	20 (13.89)	32 (22.22)	37 (26.69)	31 (21.53)	37 (25.69)	14 (9.72)	17 (11.80)

	*P* value	<0.001	<0.001	<0.001	<0.001	<0.001	0.001	<0.001	<0.001	<0.001	<0.001	<0.001	<0.001	0.013	0.013

**Table 3:** Prevalence of mean threshold change at 3000, 4000, and 6000 Hz in each job category.

Job category	Follow-up year	Threshold change			
		Negative		Positive	
		Number	Percent	Number	Percent
Control	1st	108	96.43	4	3.57
	2nd	110	98.21	2	1.79

1	1st	181	91.88	16	8.12
	2nd	148	75.13	49	24.87

2	1st	129	89.58	15	10.42
	2nd	93	64.57	51	35.43

3	1st	96	94.12	6	5.88
	2nd	87	85.29	15	14.71

## References

[1] European Agency for Safety and Health at Work (EASHW) (2000). *Monitoring the State of Occupational Safety and Health in the European Union Pilot Study*.

[2] Dunn DE, Robinowitz PM, Rosenstock L (2005). Noise. *Textbook of Clinical Occupational and Environmental Medicine*.

[3] Robinowitz PM, Rees TS, Rosenstock L (2005). Occupational hearing loss. *Clinical Occupational and Environmental Medicine*.

[4] Hong O (2005). Hearing loss among operating engineers in American construction industry. *International Archives of Occupational and Environmental Health*.

[5] Chen J-D, Tsai J-Y (2003). Hearing loss among workers at an oil refinery in Taiwan. *Archives of Environmental Health*.

[6] McBride DI, Firth HM, Herbison GP (2003). Noise exposure and hearing loss in agriculture: a survey of farmers and farm workers in the Southland region of New Zealand. *Journal of Occupational and Environmental Medicine*.

[7] National Institute for Occupational Safety and Health (NIOSH) (1996). *Criteria for a Recommended Standard, Noise Exposure- Revised Criteria*.

[8] U.S. Department of Labor, Occupational Safety and Health Administration (USDOL OSHA) Noise and Hearing Conservation. https://www.osha.gov/SLTC/noisehearingconservation/.

[9] Rösler G (1994). Progression of hearing loss caused by occupational noise. *Scandinavian Audiology*.

[10] Leensen MCJ, Van Duivenbooden JC, Dreschler WA (2011). A retrospective analysis of noise-induced hearing loss in the Dutch construction industry. *International Archives of Occupational and Environmental Health*.

[11] Smith AW (1998). The World Health Organisation and the prevention of deafness and hearing impairment caused by noise. *Noise Health*.

[12] World Health Organisation Prevention of noise induced hearing loss.

[13] Akande TM, Ologe FE (2003). Noise induced hearing loss (NIHL) in the middle belt of Nigeria. *Postgraduate Doctor Africa*.

[14] US Department of Labor (USDL), Occupational Safety and Health Administration (OSHA) (1983). Occupational noise exposure, hearing conservation amendment, final rule. *Federal Register*.

[15] Ross JAS, Macdiarmid JI, Dick FD, Watt SJ (2010). Hearing symptoms and audiometry in professional divers and offshore workers. *Occupational Medicine*.

[16] Daniell WE, Swan SS, McDaniel MM, Stebbins JG, Seixas NS, Morgan MS (2002). Noise exposure and hearing conservation practices in an industry with high incidence of workers’ compensation claims for hearing loss. *American Journal of Industrial Medicine*.

[17] Mehrparvar AH, Mirmohammadi SJ, Ghoreyshi A, Mollasadeghi A, Loukzadeh Z (2011). High-frequency audiometry: a means for early diagnosis of noise-induced hearing loss. *Noise and Health*.

[18] Baradarnfar MH, Karamifar K, Mehrparvar AH (2012). Amplitude changes in otoacoustic emissions after exposure to industrial noise. *Noise and Health*.

[19] Ologe FE, Akande TM, Olajide TG (2006). Occupational noise exposure and sensorineural hearing loss among workers of a steel rolling mill. *European Archives of Oto-Rhino-Laryngology*.

[20] Morata TC, Fiorini AC, Fischer FM (1997). Toluene-induced hearing loss among rotogravure printing workers. *Scandinavian Journal of Work, Environment and Health*.

[21] Bhattacharya SK, Tripathi SR, Kashyap S (1990). A study of heat and noise problems in a drug and pharmaceutical firm in India. *Industrial Health*.

[22] Shaikh GH (1996). Noise problem in a polyester fiber plant in Pakistan. *Industrial Health*.

[23] American National Standards Institute (2004). *Specifications for Audiometers. ANSI S3. 6-2004*.

[24] US Department of Labor (USDL) (2009). Occupational safety and exposure, hearing conservation amendment; final rule. *Federal Register*.

[25] Neitzel R, Seixas N (2005). The effectiveness of hearing protection among construction workers. *Journal of Occupational and Environmental Hygiene*.

[26] Edelson J, Neitzel R, Meischke H (2009). Predictors of hearing protection use in construction workers. *Annals of Occupational Hygiene*.

[27] Griffin SC, Neitzel R, Daniell WE, Seixas NS (2009). Indicators of hearing protection use: self-report and researcher observation. *Journal of Occupational and Environmental Hygiene*.

[28] Seixas NS, Goldman B, Sheppard L, Neitzel R, Norton S, Kujawa SG (2005). Prospective noise induced changes to hearing among construction industry apprentices. *Occupational and Environmental Medicine*.

[29] Davies H, Marion S, Teschke K (2008). The impact of hearing conservation programs on incidence of noise-induced hearing loss in Canadian workers. *American Journal of Industrial Medicine*.

[30] Rabinowitz PM, Galusha D, Dixon-Ernst C, Slade MD, Cullen MR (2007). Do ambient noise exposure levels predict hearing loss in a modern industrial cohort?. *Occupational and Environmental Medicine*.

[31] Neitzel RL, Stover B, Seixas NS (2011). Longitudinal assessment of noise exposure in a cohort of construction workers. *Annals of Occupational Hygiene*.

[32] Ahmed HO, Dennis JH, Badran O (2001). Occupational noise exposure and hearing loss of workers in two plants in eastern Saudi Arabia. *Annals of Occupational Hygiene*.

[33] Osibogun A, Igweze IA, Adeniran LO (2000). Noise-induced hearing loss among textile workers in Lagos metropolis. *The Nigerian Postgraduate Medical Journal*.

[34] Shakhatreh FM, Abdul-Baqi KJ, Turk MM (2000). Hearing loss in a textile factory. *Saudi Medical Journal*.

[35] Jhonson J, Robinson ST, Ladou J (2007). Occupational hearing loss. *Current Occupational and Environmental Medicine*.

[36] Marvel ME, Pratt DS, Marvel LH, Regan M, May JJ (1991). Occupational hearing loss in New York dairy farmers. *American Journal of Industrial Medicine*.

[37] Pirila T, Jounio-Ervasti K, Sorri M (1992). Left-right asymmetries in hearing threshold levels in three age groups of a random population. *Audiology*.

[38] Simpson TH, McDonald D, Stewart M (1993). Factors affecting laterality of standard threshold shift in occupational hearing conservation programs. *Ear and Hearing*.

[39] Suter AH (2002). *Hearing Conservation Manual*.

